# Understanding the intention to use bike-sharing system: A case study in Xi’an, China

**DOI:** 10.1371/journal.pone.0258790

**Published:** 2021-12-02

**Authors:** Xiaonan Zhang, Jianjun Wang, Xueqin Long, Weijia Li

**Affiliations:** College of Transportation Engineering, Chang’an University, Xi’an, Shaanxi, China; Univerza v Mariboru, SLOVENIA

## Abstract

Bike-sharing is widely recognized as an eco-friendly mode of transportation and seen as one of the solutions to the problem of air pollution and congestion. With the emphasis on sustainability in transportation, bike-sharing systems is an emerging topic of urban transport and sustainable mobility related research. Existing studies mainly explored the factors affecting individuals’ initial intentions to start using a shared bicycle, but few looked at the likelihood that a user would continue using one This study proposed a structural equation model with bike-sharing purchase decision involvement as independent variable, bike-sharing willingness to use as dependent variable, traveler participation and traveler perceived value as intermediary variables by introducing the concepts of purchase decision involvement, customer participation and perceived value in consumer psychology and behavior. A survey on bike-sharing users in Xi’an was conducted online and offline, and 622 effective responses were collected. The research model was tested by Amos 24.0 and the empirical results showed that All influencing factors including bike-sharing’s purchase decision-making involvement, traveler participation and traveler’s perceived value are found to be significantly and positively associated with usage intention; traveler perceived value play a chain-mediating role between bike-sharing purchase decision involvement and usage intention; bike-sharing purchase decision involvement have indirect effects on traveler perceived value through traveler participation. The results of this study enrich the current research’s in the field of sharing economy, and it is certain guiding significant for how to obtain and maintain stable customers in bicycle-sharing industry.

## 1. Introduction

Bike sharing, as a new environmental-friendly travel mode, has attracted large numbers of travelers due to its flexibility and convenience. In today’s construction of the low-carbon society, it has been promoted as an effective solution to the “last mile” problem for. At present, the research on bike-sharing mainly focuses on innovation of business management and profit models [1–3], legal system [4], influencing factors [5–7]and usage behavior [8, 9]. However, the mechanism between the influencing factors and usage intention is seldom researched. In addition, the existing literatures on the subject are mostly based on the Technology Acceptance Model (ATM) and the Theory of Planned Behavior (TPB) Model. The TAM proposed by Davis in 1989 to explain and predict the usage of information technologies [10]. TAM believes that there are external variables affecting users’ perception of usefulness and ease of use. Through the role of the two variables of user attitude and willingness to use, it indirectly affects the actual use and perceived usefulness of the system. Based on a large number of studies on technology acceptance models, it is shown that perceived ease of use positively affects perceived usefulness and attitude toward use. Perceived usefulness has a positive impact on the development of use attitude and use attitude has a positive impact on use intention [11–15].

Planned behavior theory is a social psychological theory that focuses on the determinants of individual behavior [16]. Ajzen believes that intention is the most direct and important prerequisite for people to produce concrete actions. Behavioral attitude, subjective norms and perceived behavioral control are three main variables that determine behavioral intention. The more positive the attitude, the more supportive the person, the stronger the perceived behavioral control, the greater the behavioral intention. If the attitude is not positive, the correlation variable becomes smaller [17]. Bruijn, G. J. D. et al. (2009) studied habit intensity as a moderating factor of the relationship between behavioral intention of bicycle use [18]. Sigal Kapla (2015) and other research has shown that residents of bicycle love attitude, interest in bicycle technology, good practices for bicycles, and comfort perception of bicycles and bicycle friendly countries habits of living place, traffic mode selection and the past of bicycle travel experience and interest in and use intention [19]. Acheampong, R. A. (2017) found that participants thought cycling was an easy activity. Participants felt confident that they were in control of their performance and riding ability, which had a strong effect on willingness to ride [20]. Sun L (2019) et al studied that attitude, subjective norms and personal norms ATT (attitude), PBC (perceived behavioral control) and PN (personal norms) were positively correlated with users’ civilized cycling intentions and actual behaviors [21]. Users’ willingness to ride bicycles civilly plays an intermediary role between the four influencing factors and users’ actual behavior.

The previous studies are constructed based on the relationship between perceived usefulness, perceived ease of use, attitude, subjective norm, perceived behavior control and intention. The models’ variables are mostly derived from psychology. Bike-sharing is essentially a kind of consumer choice behavior as a special commodity [22]. Generally speaking, from the perspective of consumer behavior, customers’ psychology and behavior cannot be completely separated from each. Intention was the best predictor of behavior, while the best explanatory variable for mental intentions was the past behavior [16]. The past experience and behavior were used as the explanatory variables for predicting the behavioral intention, the explanatory power of which was higher than that of attitude, subjective norms and perceived action system used as the explanatory variables [23–25]. Customers’ behaviors are dominated by their implicit psychological activities, and their behaviors will affect their psychology in turn [26]. Consuming intention is the common manifestation of customers’ psychology and behavior. Beyond that, the multi-dimensional models including cognition, emotion and behavior have attracted increasing attention [27].

The involvement and participation of customers are the basis for the formation of consumption intention psychology and behavior. Although intervention has a driving effect, it has a longer duration than motivation [28]. Customer involvement emphasizes the cognition of consciousness and behavioral input in purchase activities [29]. Previous studies have shown that the product involvement has a positive effect on brand sensituvity. Kapferer [30] and Laurent found that the level of involvement in a product can boost brand sensitivity in consumer purchases. Muratore [31] demonstrated that in children’s brand sensitivity. Lu et al. found in their study of Litchi Bay, a historic block in China, that there is a positive correlation between tourist involvement and satisfaction [32].

According to previous studies, the degree of customer involvement can influence their behavioral intention. Under the Relationship Marketing Paradigm, some empirical studies have revealed that the degree of involvement is significantly positively correlated with customers’ consumption intention. Namely, customers with high degree of involvement have strong consumption intention, and vice versa [33]. Customers with high degree of network involvement are willing to spend more time and cognitive efforts to search for information, compare differences, and consider about their choices and decisions. Furthermore, customer’s loyalty behavior will occur when a certain degree of familiarity is reached [34]. In addition, the high degree of online involvement would lead to high cognition of online shopping usability [35]. Customer participation and perceived value have a significant chain mediating effect between the purchase decision involvement and purchase intention [36]. Similarly, self-driving tourism involvement exerts a significant positive effect on behavioral intention, whereas tourism involvement plays a partially mediating role between self-driving travel motivation and behavioral intention [37]. Travelers with a high degree of involvement tend to make their choice behavior rationally, while travelers with a low degree of involvement often make choice inertially [38].

To sum up, most previous literatures study the relationships between one or two variables of customer’s involvement, participation, perceived value and consumer’s purchase intention. Yet they seldom combine the three variables to explore the influencing mechanism with intention. In addition, the previous studies mainly focus on the relevant areas of commodity consumption, while the consumption intention of bike-sharing is rarely researched. Thus, this paper introduces the related concepts of purchase decision involvement, customer participation, as well as customer perceived value, and adopts structural equation model to analyze the interactions and influence mechanism between the three variables and the usage intention. It not only expands the application scope of consumer behavior theory, but also provides bike-sharing enterprises with management and marketing strategies.

The remainder of this paper is structured as follows. Section 2 introduces the theoretical source of bike-sharing purchase decision involvement, traveler participation, traveler perceived value and the new definitions in this study. Section 3 puts forward the theoretical model and the hypothesis development. Section 4 takes Xi’an, Shaanxi province as an empirical study and presents the results of the descriptive statistics, reliability evaluation and factor analysis. Section 5. To conclude, the findings, implications, limitations and some possible directions for future research are discussed in Section 6.

## 2. Definition variables

### 2.1 Purchase decision involvement

In 1947, Sherif and Cantril first proposed the concept of involvement theory in the study of social judgement theory. The theory is mainly used to study "the problem of personal attitude in social events". However, the definitions are various in other fields. The definition proposed by Zaichkowsky in 1985 has been widely accepted by scholars, i.e. " Decision involvement means the degree of correlation to the target perception of individual’s internal needs, value and interest" [39]. The concept better represents the involvement of an internal psychological state and intention with the resulting consumer behavior. Purchase decision involvement refers to the degree to which consumers are concerned about purchase decisions. It can be divided into product involvement, advertisement involvement and purchase decision involvement according to the object involvement of influence. Mittal clearly distinguished purchase decision involvement from product involvement, and defined purchase decision involvement as consumers’ interest and degree of concern. At the same time, four consumer purchase decision involvement scales(PII, CPI, PDI, FCB) were developed through empirical methods [40].

Based on the existing studies, Bike-sharing purchase decision refers primarily to the degree of travelers ‘ concern to bike-sharing in this paper. It is a temporary concern for decision-making on bike-sharing choice under the combined effect of user’s internal demand such as own needs, interests, values and external situations such as social environment. The design scale is mainly considered from two dimensions: personal values and community attributes.

### 2. 2 Customer participation

Customer participation is usually defined as behavior in the process of service production or delivery [41, 42]. Scholars have used different methods to measure the dimension of customer participation based on different perspectives. In 1985, Fisk and Sialkot defined customer participation as consumer effort and involvement (including mental and physical participation). Sialkot believed that customer participation can be divided into three aspects input: mental input, physical input and emotional input. The cognitive ability of customers to obtain and process information is regarded as mental input, customers’ consumption of tangible objects and physical fitness are regarded as physical input, and customers’ emotional intensity is regarded as emotional input [43]. Kelley et al. (1990) [44] believed that certain service transactions must be completed by providing certain information or making efforts. Cermak, File, and Prince [45] pointed out that customer participation is a behavior related to service delivery, and was related with service specifications and service delivery. It is a customer’s personal activity, the importance of single association or commodity to consumers is customer input.

In this paper, customer participation means traveler participation. It refers to the specific behavior of travelers spending time, energy, intelligence and emotion when using bike-sharing. It can be described in detail as: whether to download bike-sharing Apps, buy a bike-sharing rental card, pay attention to preferential activities, and such associated behaviors.

### 2. 3 Perceived value

Perceived value refers to subjective evaluation of the utility of a product or service, which is obtained by subtracting the cost of acquiring the product or service from the perceived benefit of customer. In a general sense, perceived value is different from the objective value of the product or service, which reflects the specific cognitive value of customer [46]. Customer perceived value (CPV) is usually considered as a multi-dimensional concept. According to the theory of consumption value, SHETH et al. [47] put forward the five customer perceived value dimension, which included the functional value related to the practical function of product, the social value related to social image, the emotional value related to feeling of customer, the cognitive value related to customer’s curiosity about new things, and the situational value in a specific environment. The contribution of this theory lies in its practicality and conciseness. It is easier for people to understand the connotation of customer perceived value through dimensionality division. SWEENEY et al. [48] believed that the price and quality are both functional value factors, but their contribution to customer perceived value should be measured separately. Therefore, on the basis of SHETH’s theory, the function value is further divided into two sub-dimensions, namely quality factor and price factor. The PERVAL scale of customer perceived value is verified through empirical research on durable goods, including function value quality factor, function value price factor, social value and emotional value. Liu et al. divided consumers’ perception value of electric vehicles into functional value, emotional value, social value and cognitive value [49].

To sum up, the perceived value in this study refers to the riders’ subjective evaluation of the emotional value, functional value and safety value based on the trade-off between travel gains and losses.

## 3. Hypothesis development and conceptual model

### 3.1 hypothesis

#### 3.1.1 Bike-sharing purchase decision involvement and traveler participation

Involvement is a psychological state variable, focusing on the individual’s attitude, such as the degree of attention, self-relevance, etc. Engagement is a behavioral variable, focusing on customer behavior. Involvement is the antecedent variable of information search, which belongs to customer participation behavior [50]. Customers with high network involvement are more willing to give more time and cognitive effort to search for information, compare differences and make decisions. Consumer involvement is an antecedent variable of customer participation, which has a positive impact on the four dimensions of customer participation, including preparation, information exchange, cooperative behavior and interpersonal interaction [51]. This paper attempts to analyze and verify the effect of bike-sharing purchase decision involvement on traveler participation and proposes the following hypothesis:

H1: Bike-sharing purchase decision involvement has a positive impact on traveler participation.

#### 3.1.2 Bike-sharing purchase decision involvement, traveler perceived value and usage intention

An empirical study showed that tourists involvement has a positive impact on perceived value of tourist attractions and plays a mediating role in the influence of motivation on tourists’ perceived value [52]. Campbell believed that the higher the consumer’s product involvement was, the stronger the willingness to purchase was [53]. In the field of organic food consumption in Taiwan, it has been verified that, consumer involvement has positive impact on purchase intention, and involvement played an intermediary role between purchase motivation and purchase intention [54]. The same conclusion had been obtained in the field of new energy vehicles [55]. Therefore, the hypothesis are proposed:

H2: Bike-sharing purchase decision involvement has a positive impact on traveler perceived value.
H3: Bike-sharing purchase decision involvement has a positive impact on intention of bike-sharing usage.

#### 3.1.3 Traveler participation and traveler perceived value

The impact of customer participation on perceived value has been confirmed by many scholars. Under the condition of service success, the higher the degree of customer participation is, the greater the perceived value has [56]. Social network users‘ participation behavior such as browsing, interaction and creation has a significant positive impact on users’ perceived value [57]. Therefore, the following hypothesis has been proposed in this paper:

H4: Traveler participation has a positive impact on traveler perceived value.

#### 3.1.4 Traveler participation and intention of bike-sharing usage

An empirical study on real estate marketing, customer participation has a significant positive impact on purchase intention [58]. In a B2B business model of the building materials and design industry, customer participation had a significant positive effect on traveler perceived value and an indirect effect on behavioral intention [59]. A study on consumption behavior of e-coupons behavior verified traveler perceived value plays a mediating effect on customer participation and purchase intention [60]. Therefore, the following hypothesis is proposed:

H5: Traveler participation has a positive impact on intention of bike-sharing usage.

#### 3.1.5 Traveler perceived value and intention of bike-sharing usage

Many studies have shown that perceived value is an important antecedent variable of behavior intention. A study on the Malaysian housing market, it was found that functional value, social value, relationship value, situational value and cognitive value have significant positive effects on purchase intention [61]. In the study of revisiting tourist destinations, when a tourist with a high perceived value in a tourist destinations tend to revisit or actively recommend behaviors in the future [62]. Customer perceived value has a significant impact on consumer behavioral intention in the sharing economy [63]. There is a significant positive correlation between consumer perceived values and purchase intentions in online shopping. Taking clothing brands as the research object, traveler perceived value, network interaction and brand relationship have significant positive effects on consumer behavioral intention [64]. Therefore, the following hypothesis is proposed:

H6: Traveler perceived value has a positive impact on the intention of bike-sharing usage.

#### 3.1.6 The chain-mediating role of traveler perceived value and traveler participation

Traveler purchase decision involvement, participation, perceived value and intention of bike-sharing usage conform to the time sequence of the consumer decision-making process model. Involvement is the prerequisite and starting point of the purchase decision-making process, the level of purchase decision involvement will cause differences in a series of subsequent behaviors [65]. That is, the higher involvement in the decision-making of travel mode selection, the clearer the perceived value of travelers have, it can promote the willingness to use shared bicycles. Therefore, four variables of bike-sharing purchase decision involvement, traveler participation, traveler perceived value and intention of bike-sharing usage are included in this study. On the basis of Hypotheses H1 to H6, the following hypothesis are proposed:

H7: Bike-sharing purchase decision Involvement affects usage intention through the mediating role of traveler participation and traveler perceived value.
H8: bike-sharing purchase decision involvement have indirect effects on traveler perceived value through traveler participation.

### 3.2 Structural equation model

The Structural Equation Model(SEM) is a quantitative research method in contemporary social, psychological, and behavioral sciences. It is a method of establishing, estimating and testing causality. It includes methods such as multiple regression, factor analysis, and path analysis. SEM can clearly analyze the effect of individual indicators on the overall and the interrelationship between individual indicators.

SEM includes two parts: measurement model and structural model. Measurement model can be expressed as:

$$X=\Lambda_{X}*\xi +\delta$$ (1)

$$y=\Lambda_{y}*\eta +\varepsilon$$ (2)

Formula (1) is the measurement model of exogenous variables. Formula (2) is the measurement model of endogenous variables.

Structural model can be expressed as:

$$y=\Lambda_{y}*\eta +\varepsilon$$ (3)

Formula (3) means the structural relationship of potential exogenous variables and potential endogenous variables.

Where, *X* represents the observable variable vector, *Λ*_*X*_ and *Λ*_*y*_ represent the factor load matrix, *ξ*, *η* represent the vector of latent variables (common factors), and *δ ε* represent the vector of errors (unique factors).

### 3.3 Conceptual model

Based on the above hypotheses, this paper proposes a bike-sharing usage intention model based on SEM S1 Fig including the independent variables bike-sharing purchase decision involvement, the dependent variable usage intention, and the mediating variables traveler participation and traveler perceived value.

## 4. Materials and methods

### 4.1 Participants and procedure

A 36-item questionnaire was designed to collect data from a sample of residents of Xi’an. The questionnaire consisted of two sections: the first part collected sociodemographic of the interviewees, including gender, age, occupation, income, education, private car ownership and other basic characteristics information. The second part measured residents’ willingness to use bike-sharing by 5-point Likert scale, which includes some descriptive views of residents’ personal values, community influence, perceived value, etc. while the measurement of personal values, community influence and usage intention was adapted from the literature [10, 66, 67], traveler participation and perceived value was based on the scales by Liang m et al [68–70].

Before the formal investigation, we conducted an online survey with written informed consent, obtaining 203 questionnaires in the pre-survey. After conducting a content analysis of the questionnaires and consulting with survey experts, certain items was modified and improved within the questionnaire, a formal questionnaire comprised 6 variables and 24 measurement items. The questionnaire statements for measuring usage factors are shown in Table 1.

**Table 1:** Questionnaire statements for measuring usage factors.

Factors	Dimension	Items
**bike-sharing purchase decision involvement**	1–1	I often care about environmental issues
	1–2	My friends think it’s fashionable to use bike-sharing
	1–3	Friends, relatives and neighbours often use bike-sharing
	1–4	I try to have a low-carbon life
	1–5	I often get information from news, magazines, social media and other networks about encouraging people to use bike-sharing
**Traveler participation**	2–1	I understand the operating procedures and charging rules of bike-sharing
	2–2	I have browsed and understood the relevant contents and functions about bike-sharing App software
	2–3	I have used bike-sharing discount card/monthly card/season card/annual card
	2–4	I often pay attention to the preferential activities in the bike-sharing App
**Perceived emotional value**	3–1	Enjoy the fresh air of avenue
	3–2	Using bike-sharing can relieve stress and make people feel happy
	3–3	Using bike-sharing can strengthen the body
	3–4	Ensure the accuracy of travel time, which gives me a sense of security
	3–5	Choosing green travel is more important than the convenience brought by car
**Perceived functional value**	4–1	The App is easy to download and use
	4–2	Customer service can quickly solve the problem of customer feedback
	4–3	Ride comfort
	4–4	Reasonable number and even distribution
**Perceived security value**	5–1	Security of payment
	5–2	Security of personal information
	5–3	Personal safety when riding
**Usage Intention**	6–1	I will continue to use shared bikes
	6–2	I will often use shared bikes
	6–3	I am willing to encourage/persuade people around me to use shared bikes

It should be noted that, in the first page of the questionnaire, we introduced our research purpose and secured their written informed consent to participate in this academic research. Non-adult participants were allowed to answer this questionnaire only after getting their parent or guardian’s written informed consent. The Chang’an University Ethics Committee approved the protocol and informed consent forms for this study.

### 4.2 Data collection

The questionnaires were collected though the combination of online and offline survey. The online survey questionnaires are distributed by posting a microtask on the www.wjx.cn, one of the largest free research platforms in China. The offline paper-based survey questionnaires, we randomly distributed them to the residents in Xi’an, For the specific survey site, we divided the Xi’an study region into seven urban districts(Beilin, Lianhu, Baqiao, Weiyang, Yanta, Xincheng, Chang’an) and 35 collection blocks. Both the online and offline respondents received 2RMB each for participation.

A total of 650 questionnaires were collected, 500 field survey questionnaires and 150 online questionnaires, yielding 622 valid questionnaires, with an effective response rate of 95.7% (see Table 1). The demographic characteristics of the sample, including gender, age, occupation, education, and income, were recorded. Of the respondents, 52.1% (n = 324) were male and the others female (n = 298). The age grouping with the greatest number of responses was 19–30years of age(47.4%),followedby31–45years of age(44.8%). As for education and income, the greater part of the participants hold an undergraduate degree (65.9%) and 13.5% were graduate students. Further, the largest response group reported a monthly income below CNY3000 (41.6%), and the next group represented 26.4% reported an income 3001–6000 yuan, high income group(monthly income above CNY10000) accounts for 10.0%. Our sample’s age distribution is consistent with the results of 2018 China shared Bicycle Industry Research Report [71]. Thus, our sample can be representative of the population of interest. The respondents’ characteristics can be seen in Table 2.

**Table 2:** Descriptive statistics of research samples, n = 622.

Indicators Question options		n	Percentage (%)
**Gender**	Male	324	52.1
	Female	298	47.9
**Age group**	Under 18	6	1.0
	19–30 years old	295	47.4
	31–45 years old	275	44.8
	over46	42	6.8
**Occupation**	student	218	35.0
	Civil servant	19	3.1
	Personnel of enterprises and institutions	186	29.9
	Clerk	102	16.4
	Self employed	31	5.0
	Unemployed and other	66	10.6
**Monthly income**	<3000 yuan	259	41.6
	3001–6000 yuan	164	26.4
	6001–10000 yuan	137	22.0
	>10000 yuan	62	10.0
**Education**	High school and below	76	12.2
	Technical secondary school or college	84	13.5
	Undergraduate	410	65.9
	Master’s degree or above	52	8.4
**Weekly usage frequency**	Less than 2 times	451	72.5
	3–6 times	121	19.5
	7–10 times	28	4.5
	More than 10 times	22	3.5
**Travel distance**	≤3 km	537	86.3
	4–8 km	70	11.3
	>8 km	15	2.4
**Frequency of different travel purposes**	Commuting/school	200	32.2
	Shopping/ Eating	34	5.5
	Leisure time	161	25.8
	Connecting with other modes of transportation	149	24.0
	Handling affairs	78	12.5

### 4.3 Reliability and validity

SPSS version26.0 software was used to test the quality of the data. The entire questionnaire’s Cronbach’s α reliability coefficient was 0.927, While the four domains, including bike-sharing purchase decision involvement, traveler participation, traveler perceived value and usage intention, were 0.852, 0.821, 0.898 and 0.871, respectively, in which the perceived emotional value, perceived functional value and security value were 0.887, 0.810 and 0.835, respectively. All of the subscales met the minimum standards of reliability (Cronbach’s alpha coefficient >.70) [72]. The result showed a high degree of internal consistency and test-retest reliability, as shown in Table 3.

**Table 3:** Reliability analysis of the questionnaire.

Variable	Dimension	Items	Cronbach’s alpha
**Bike-sharing purchase decision involvement**	/	5	0.852
**Traveler participation**	/	4	0.821
**Traveler perceived value**	Perceived emotional value	5	0.887
	Perceived functional value	4	0.810
	Perceived security value	3	0.835
**Usage intention**	/	3	0.871
**Overall reliability of the questionnaire**	/	24	0.927

An Exploratory factor analysis (EFA) was performed with the results showing clear evidence of a 6-factor structure with eigen values over Kaiser’s criterion of 1.00. The newly generated 6-factor model collectively explained 70.0% of the variance, the load of each item on its factor is much more than 0.5, while the load on other factors is much less than 0.5, this shows each factor is unique [73]. The Kaiser-Meyer-Olkin (KMO) value was 0.916 and this measure verified the sampling adequacy for the analysis. Bartlett’s test of sphericity indicated that the correlations between items were sufficiently large for the EFA analysis (p < 0.001). The scree plot test was used to determine the number of factors to retain and rotate with the test results confirming the appropriateness of a 6-factor solution. The EFA yielded an 24-item measure with a 6-factor structure. The 6 factors were labeled as Bike-sharing purchase decision involvement, Traveler participation, Perceived emotional value, Perceived functional value, Perceived security value and Usage intention. Reliability of the items in each of the four TRAS subscales was tested using reliability analysis. Table 4 shows the factor loadings after rotation using the Promax method.

**Table 4:** Factor loadings.

Item	Factor Loadings					
	F1	F2	F3	F4	F5	F6
	(Perceived emotional value)	(Bike-sharing purchase decision involvement)	(Traveler participation)	(Perceived functional value)	(Perceived security value)	(Usage intention)
3–3	**.834**	.175	.112	.129	.231	.107
3–2	**.821**	.179	.031	.129	.163	.128
3–1	**.764**	.200	.138	.136	.206	.136
3–5	**.702**	.247	.179	.121	.142	.077
3–4	**.654**	.085	.159	.311	.021	.292
1–3	.118	**.779**	.169	.031	.133	.057
1–4	.191	**.765**	.205	.048	.135	.036
1–2	.169	**.747**	.018	.118	.012	.191
1–1	.118	**.741**	.048	.197	-.057	.163
1–5	.189	**.684**	.170	.166	.090	.192
5–4	.137	.198	**.786**	.058	.209	.031
5–3	.096	.027	**.766**	.022	.182	.157
5–1	.130	.138	**.729**	.250	.039	.225
5–2	.126	.234	**.723**	.244	.027	.141
2–2	.087	.078	.040	**.789**	.133	.074
2–3	.132	.147	.198	**.707**	.253	.002
2–1	.242	.170	.165	**.691**	.105	.233
2–4	.240	.156	.160	**.670**	.271	.098
4–2	.222	.080	.163	.228	**.816**	.076
4–3	.211	.113	.221	.243	**.760**	.108
4–1	.230	.061	.084	.243	**.692**	.250
5–5	.236	.241	.227	.141	.161	**.818**
5–6	.214	.253	.222	.153	.167	**.817**
5–7	.264	.384	.257	.112	.224	**.524**

Table 4. Factor Loadings for Malay Version of the TRAS from Exploratory Factor Analysis Using the Promax Method (N = 622)

## 5. Estimated result

### 5.1 Direct effect test

AMOS structural equation model was used to verify the hypothesis. The common parameter estimation method of structural equation model is maximum likelihood or generalized least square method [74]. Both methods require the data is subject to multivariate normal distribution. Therefore, the normal distribution of scale data should be checked before model detection. The results of normal distribution test showed that the multivariate kurtosis value of the sample was 68.293, as shown in Table 5. This indicates that the sample data are significantly non-normal.

**Table 5:** Statistics for assessing the normality of the observed variables in the model.

Variable	min	max	skew	c.r.	kurtosis	c.r.
1–4	1.000	5.000	-.055	-.564	-.382	-1.942
2–4	1.000	5.000	-.013	-.135	.472	2.405
3–1	1.000	5.000	-.644	-6.557	.357	1.815
1–5	1.000	5.000	-.377	-3.840	-.250	-1.271
2–3	1.000	5.000	-.285	-2.901	.468	2.381
2–1	1.000	5.000	-.289	-2.941	.387	1.969
2–2	1.000	5.000	-.213	-2.166	.095	.484
5–5	1.000	5.000	-.477	-4.862	.161	.822
5–6	1.000	5.000	-.532	-5.419	.223	1.135
5–7	1.000	5.000	-.297	-3.025	-.074	-.375
5–3	1.000	5.000	-.458	-4.664	-.611	-3.108
5–2	1.000	5.000	-.228	-2.321	.017	.087
5–1	1.000	5.000	-.216	-2.204	-.121	-.616
4–3	1.000	5.000	.015	.153	.309	1.571
4–2	1.000	5.000	-.148	-1.510	.326	1.660
4–1	1.000	5.000	-.281	-2.862	.465	2.369
3–4	1.000	5.000	-.371	-3.774	-.122	-.622
3–3	1.000	5.000	-.541	-5.504	.311	1.584
3–2	1.000	5.000	-.683	-6.950	.621	3.164
1–3	1.000	5.000	-.351	-3.573	-.528	-2.686
1–2	1.000	5.000	-1.336	-13.601	1.816	9.243
1–1	1.000	5.000	-.287	-2.921	.130	.660
Multivariate					177.975	68.295

One of the methods to solve the non-normal distribution of sample data is bootstrap method [75]. Bollen-stine bootstrapping procedure can be used to estimate the standard error and correct the deviation of model fitting data [76]. In addition, Nevitt and Hancock suggest that boot value analysis is more suitable for samples with a sample size of over 200 [77]. The empirical study focused on 622 samples tested by the model and the non-normal distribution of samples. Therefore, Bollen-Stine bootstrapping procedure was adopted in this study. The Table 6 and S2 Fig depicts the results of hypotheses test. all of the path coefficients had acceptable statistical significance level. Hence, H1, H2, H3,H4,H5 and H6 were supported.

**Table 6:** Preliminary hypothesis test results.

Hypothesis	Relationship	Standardized path coefficient	T value	P value	Conclusion
**Hypothesis H1**	Bike-sharing purchase decision involvement→ traveler participation	0.496	10.363	***	support
**Hypothesis H2**	Bike-sharing purchase decision involvement→ Traveler perceived value	0.364	6.433	***	support
**Hypothesis H3**	Bike-sharing purchase decision involvement→ Usage intention	0.251	5.029	***	support
**Hypothesis H4**	Traveler participation →Traveler perceived value	0.446	8.213	***	support
**Hypothesis H5**	Traveler participation →usage intention	0.187	3.670	***	support
**Hypothesis H6**	Traveler perceived value →Usage intention	0.418	6.492	***	support

Note: *** = significant at the 0.001 level.

It can be seen from Table 7 the chi square ratio is 3.866, which is higher than the desired cutoff value of 3.000. The root mean squared error of approximation (RMSEA) is 0.068, which is lower than 0.080. The Normed Fit Index (NFI) is 0.891, and the Comparative Fit Index (CFI) is 0.876, which is lower than 0.9. The Tucker-Lewis Index(TLI) is 0.905, which are greater than 0.900. To summarize, the structural model should be improved.

**Table 7:** Preliminary model fitness index.

Index	Model fitness index	Standard value	Conclusion
**Chi-square value/degrees of freedom**	3.866	>3.000	refuse
**RMSEA**	0.068	<0.08	accept
**NFI**	0.891	>0.9	refuse
**CFI**	0.876	>0.9	refuse
**TLI**	0.905	>0.9	accept

According to the MI index, four measuring items, including Q2-4, Q3-4, Q3-5 and Q4-4 were deleted. The optimized chi square ratio is 2.894, which is below the desired cutoff value of 3.000. The root mean squared error of approximation (RMSEA) is 0.055, which is lower than 0.080. The Normed Fit Index (NFI) is 0.932, and the Comparative Fit Index (CFI) is 0.954, and the Tucker-Lewis Index(TLI) is 0.946, both of which are greater than 0.900. To summarize, the structural model has a good fit. The final model path diagram and relevant index are shown in S3 Fig and Table 8.

**Table 8:** Final model fitness index.

Index	Model fitness index	Standard (ideal value)	Conclusion
**Chi-square value/degrees of freedom**	2.984	>3.000	accept
**RMSEA**	0.055	<0.08	accept
**NFI**	0.932	>0.9	accept
**CFI**	0.954	>0.9	accept
**TLI**	0.946	>0.9	accept

The Table 9 depicts the results of hypotheses test. all of the path coefficients had acceptable statistical significance level. The p-values of the effects of bike-sharing purchase decision involvement on traveler participation, traveler perceived value, usage intention, the effects of traveler participation on Traveler perceived value and the effects of traveler perceived value on usage intention are all less than 0.001. Hence, H1, H2, H3,H4 and H6 were supported. Traveler participation showed lower significance on the Usage intention (p<0.05), but it also can be said that this effect was significant. That is to say, H5 was supported.

**Table 9:** Final model hypothesis test results.

Hypothesis	Relationship	Standardized path coefficient	T value	P value	Conclusion
**Hypothesis H1**	Bike-sharing purchase decision involvement→ Traveler participation	0.504	9.778	***	accept
**Hypothesis H2**	Bike-sharing purchase decision involvement→ Traveler perceived value	0.395	6.237	***	accept
**Hypothesis H3**	Bike-sharing purchase decision involvement→ Usage intention	0.294	5.250	***	accept
**Hypothesis H4**	Traveler participation→ Traveler perceived value	0.408	7.386	***	accept
**Hypothesis H5**	Traveler participation→Usage intention	0.164	3.287	**	accept
**Hypothesis H6**	Traveler perceived value→Usage intention	0.398	6.143	***	accept

Note: ** = significant at the 0.05 level,

*** = significant at the 0.001 level.

### 5.2 Intermediary effect test

Next, the mediation effect of traveler perceived value was analyzed in the SEM model and present the result in Table 10. Among several methods of testing the mediation effect, the causal steps approach and the Sobel test have been used widely but have also been criticized by researchers [78, 79]. So, we adopt a bootstrapping method that results in more accurate confidence intervals for indirect effects [80]. We use AMOS 24 to calculate 5000 bootstrap samples. If zero is not between the lower and upper bound of the bias-corrected percentile and percentile, the mediation effect is shown to be significant at a confidence interval of 95%. As presented in Table 9, bike-sharing purchase decision involvement have indirect effects on usage intention through traveler perceived value, meanwhile, bike-sharing purchase decision involvement have indirect effects on traveler perceived value through traveler participation, and their indirect effect were 0.286 and 0.141 respectively, the 95% confidence interval for the bias-corrected percentile and percentile does not contain zero. So, hypotheses H7 and H8 were supported. The direct effect of bike-sharing purchase decision involvement have on usage intention is 0.262 and the direct effect of bike-sharing purchase decision involvement have on traveler perceived value is 0.271, both of the 95% confidence interval for the bias-corrected percentile and percentile does not contain zero. In summary, the above results indicated that traveler perceived value and traveler participation played partial mediating roles.

**Table 10:** Mediating effects of traveler participation and traveler perceived value.

Variable	Effect	Estimate	BC 95% CI			Percentile 95% CI		
			Lower	Upper	P	Lower	Upper	P
Bike-sharing purchase decision involvement →Traveler participation	Total Effects	0.548	0.416	0.688	***	0.418	0.692	***
	Direct Effects	0.262	0.111	0.436	***	0.105	0.431	***
	Indirect Effects	0.286	0.177	0.422	***	0.177	0.421	***
Bike-sharing purchase decision involvement →Traveler perceived value	Total Effects	0.412	0.284	0.556	***	0.284	0.556	***
	Direct Effects	0.271	0.150	0.421	***	0.152	0.181	***
	Indirect Effects	0.141	0.092	0.210	***	0.086	0.203	***

Note: *** = significant at the 0.001 level

## 6. Discussion

The empirical results showed that the twenty main questionnaire items from bike-sharing purchase decision involvement, traveler participation and traveler perceived value had the influence on usage intention to different degrees. The specific conclusions were obtained as follows: All influencing factors including bike-sharing’s purchase decision-making involvement, traveler participation and traveler’s perceived value are found to be significantly and positively associated with usage intention. That is, bike-sharing’s purchase decision-making involvement, traveler participation and traveler’s perceived value are significant predictors of usage intention. Hence, Hypotheses H1, H2, H3, H4, H5 and H6 are supported.

Seen from the degree of influence, traveler perceived value had the greatest effect on usage intention (in Table 9), it reflected the fitness, leisure, comfort and safety aspects of bike-sharing had played a great role in intention of bike-sharing usage. It is also one of the reasons for the popularity of bike-sharing and is consistent with existing research conclusions [81]. Then, Bicycle-sharing purchase decision involvement not only has a direct impact on willingness to use, but also has an indirect impact on willingness to use through intermediary variables such as traveler participation and traveler perceived value. Among the dual influences, the indirect effect is dominant, followed by the direct effect, which reflects the usage intention of bike-sharing is positively influenced by personal values, reference groups and others, and the social impact of bike-sharing that conforms to mainstream values of modern energy conservation, environmental protection and low-carbon travel will result in herd consumption. This finding is consistent with previous studies on personal values and community influence [82]. Finally, the effect of traveler participation on usage intention reflects the bike-sharing as a new business model innovation have attraction with different levels for travelers due to different operating procedures, membership systems, preferential information and other activities of operators, which is consistent with the study of the willingness to use e-coupons [83].

The main contributions of this study come from three aspects: first of all, the influential factors of bike-sharing usage intention have been revealed from the three-dimensional perspective of bike-sharing purchase decision involvement, traveler participation and traveler perceived value, which provides a more comprehensive perspective on psychology and behavior for bike-sharing usage. Secondly, this paper proposes a theoretical model of bike-sharing usage intention based on the consumer decision-making process model that enriches empirical research on the bicycle-sharing industry and broadens the scope of application of consumer behavior theory. Finally, these results can guide the management practices of bike-sharing enterprises to improve the sustainable utilization rate by improving the user experience through convenience, environmental protection, low price, etc.

This study also has some limitations, and other factors such as residents’ travel characteristics, weather, as well as the built environment should be considered in future research. Furthermore, the sample size can be expanded and the groups can be divided, which will help bike-sharing enterprises to understand intention differences between different groups and put forward feasible strategy and targeted tactics.

## Supporting information

S1 Fig Conceptual model. (TIF)

S2 Fig Preliminary model path diagram. (TIF)

S3 Fig Final model path diagram. (TIF)

S1 Data (XLS)
