# Supplementary figures and images for: Understanding the intention to use bike-sharing system: A case study in Xi’an, China
Source: PLoS One. 2021 Dec 2;16(12):e0258790. doi: 10.1371/journal.pone.0258790 (PMC8638983; doi:10.1371/journal.pone.0258790)

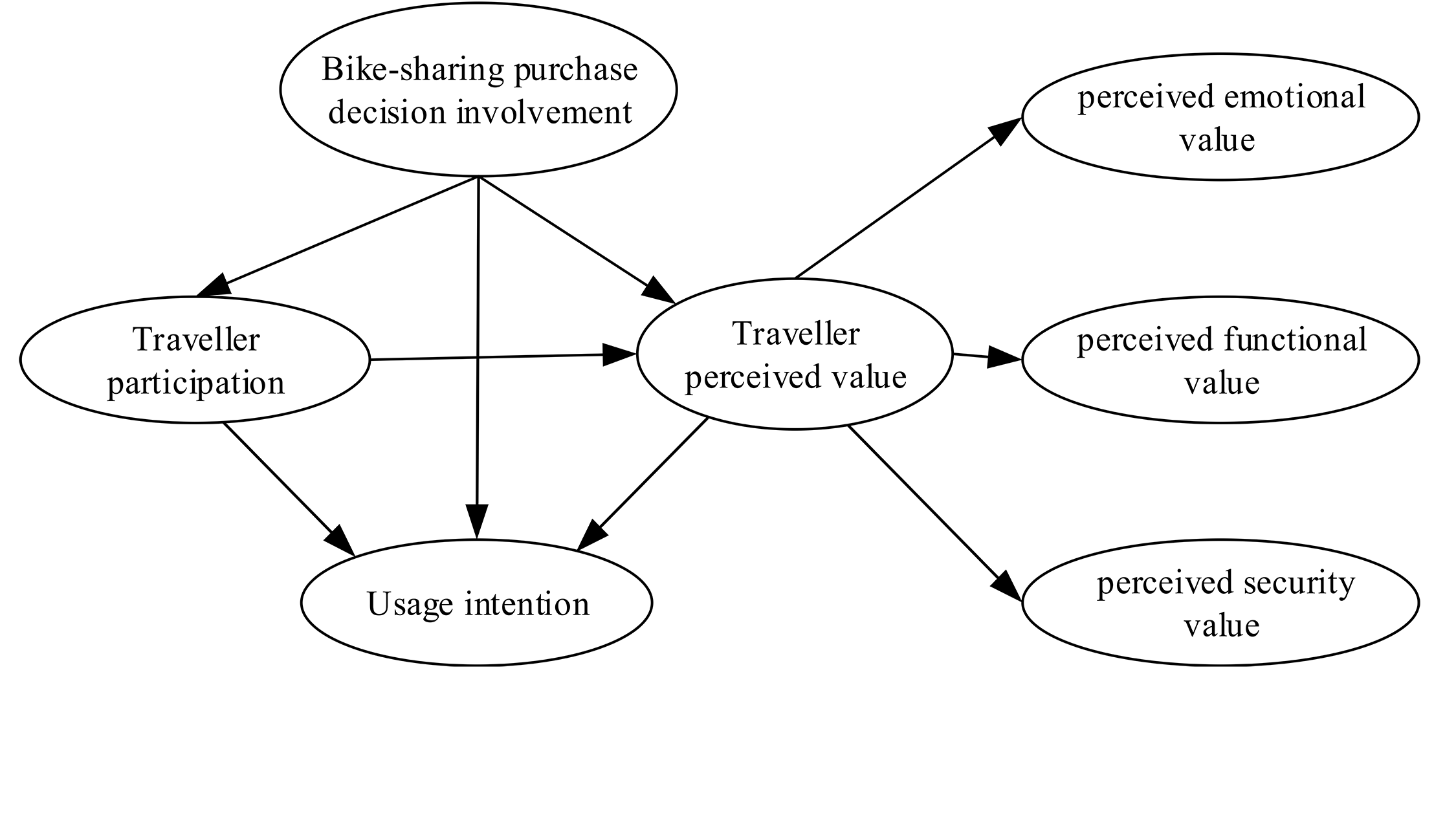

Supplement: S1 Fig — (TIF) [file pone.0258790.s001.tif]

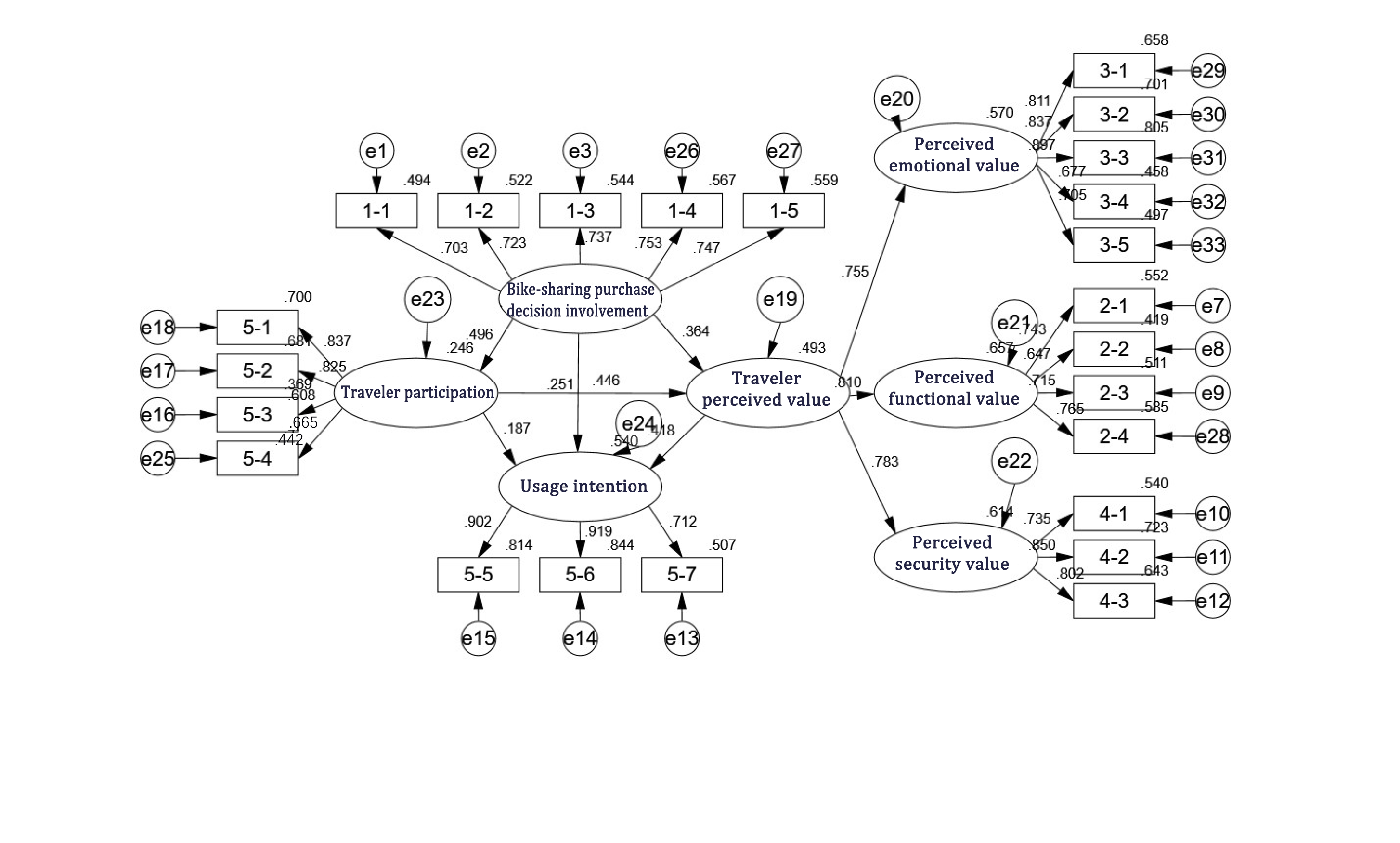

Supplement: S2 Fig — (TIF) [file pone.0258790.s002.tif]

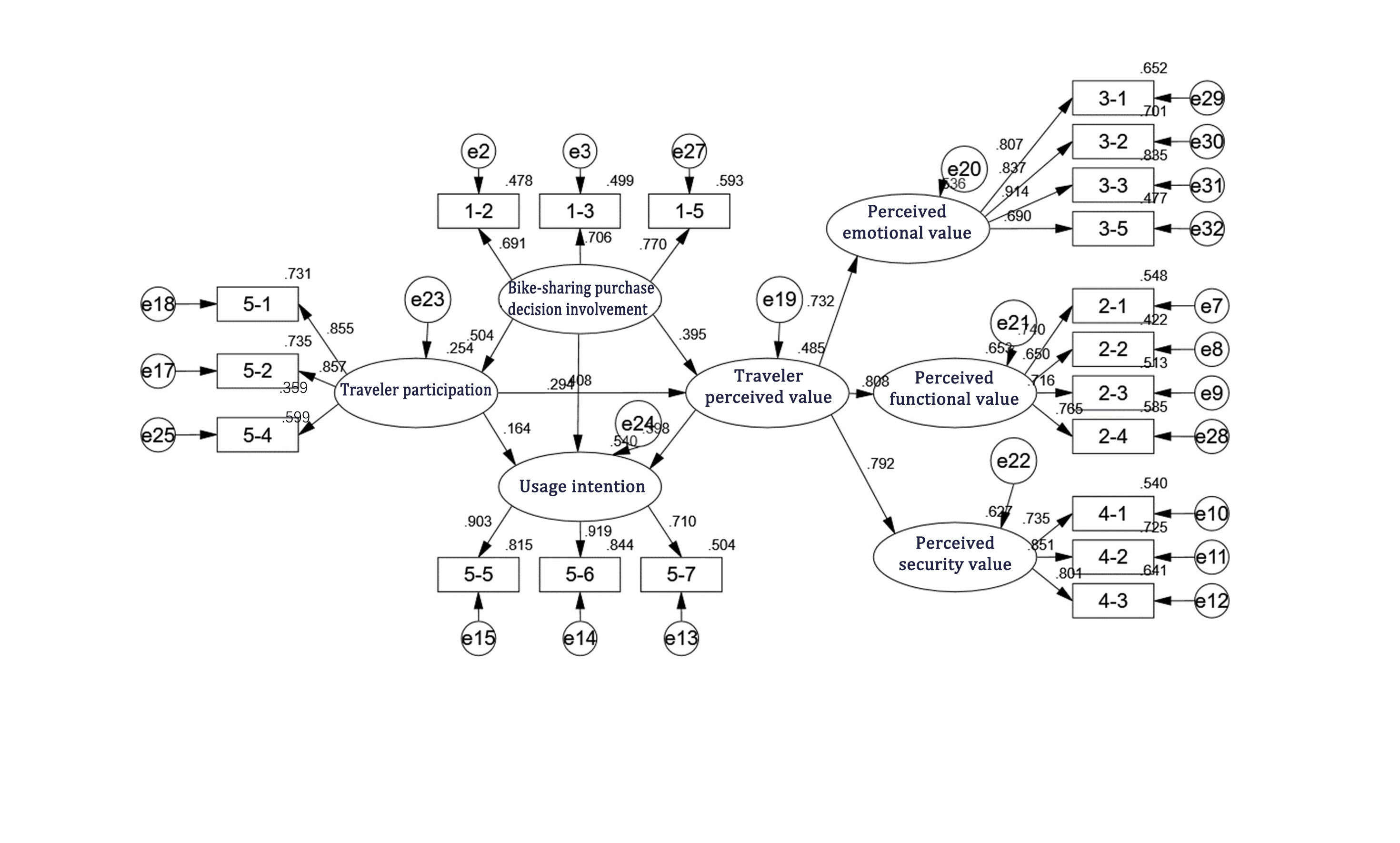

Supplement: S3 Fig — (TIF) [file pone.0258790.s003.tif]
